# Efficacy and safety of CD19 CAR T constructed with a new anti-CD19 chimeric antigen receptor in relapsed or refractory acute lymphoblastic leukemia

**DOI:** 10.1186/s13045-020-00953-8

**Published:** 2020-09-07

**Authors:** Runxia Gu, Fang Liu, Dehui Zou, Yingxi Xu, Yang Lu, Bingcheng Liu, Wei Liu, Xiaojuan Chen, Kaiqi Liu, Ye Guo, Xiaoyuan Gong, Rui Lv, Xia Chen, Chunlin Zhou, Mengjun Zhong, Huijun Wang, Hui Wei, Yingchang Mi, Lugui Qiu, Lulu Lv, Min Wang, Ying Wang, Xiaofan Zhu, Jianxiang Wang

**Affiliations:** 1 State Key Laboratory of Experimental Hematology, National Clinical Research Center for Blood Diseases, Institute of Hematology & Blood Diseases Hospital, Chinese Academy of Medical Sciences & Peking Union Medical College, Tianjin, 300020 China; 2 Juventas Cell Therapy Ltd., Tianjin, 300384 China

**Keywords:** Chimeric antigen receptor-modified T cell, Single-chain variable fragment, Acute lymphoblastic leukemia, HI19α

## Abstract

**Background:**

Recent evidence suggests that resistance to CD19 chimeric antigen receptor (CAR)-modified T cell therapy may be due to the presence of CD19 isoforms that lose binding to the single-chain variable fragment (scFv) in current use. As such, further investigation of CARs recognize different epitopes of CD19 antigen may be necessary.

**Methods:**

We generated a new CD19 CAR T (HI19α-4-1BB-ζ CAR T, or CNCT19) that includes an scFv that interacts with an epitope of the human CD19 antigen that can be distinguished from that recognized by the current FMC63 clone. A pilot study was undertaken to assess the safety and feasibility of CNCT19-based therapy in both pediatric and adult patients with relapsed/refractory acute lymphoblastic leukemia (R/R B-ALL).

**Results:**

Data from our study suggested that 90% of the 20 patients treated with infusions of CNCT19 cells reached complete remission or complete remission with incomplete count recovery (CR/CRi) within 28 days. The CR/CRi rate was 82% when we took into account the fully enrolled 22 patients in an intention-to-treat analysis. Of note, extramedullary leukemia disease of two relapsed patients disappeared completely after CNCT19 cell infusion. After a median follow-up of 10.09 months (range, 0.49–24.02 months), the median overall survival and relapse-free survival for the 20 patients treated with CNCT19 cells was 12.91 months (95% confidence interval [CI], 7.74–18.08 months) and 6.93 months (95% CI, 3.13–10.73 months), respectively. Differences with respect to immune profiles associated with a long-term response following CAR T cell therapy were also addressed. Our results revealed that a relatively low percentage of CD8^+^ naïve T cells was an independent factor associated with a shorter period of relapse-free survival (*p* = 0.012, 95% CI, 0.017–0.601).

**Conclusions:**

The results presented in this study indicate that CNCT19 cells have potent anti-leukemic activities in patients with R/R B-ALL. Furthermore, our findings suggest that the percentage of CD8^+^ naïve T cells may be a useful biomarker to predict the long-term prognosis for patients undergoing CAR T cell therapy.

**Trial registration:**

ClinicalTrials.gov: NCT02975687; registered 29 November, 2016. https://clinicaltrials.gov/ct2/keydates/NCT02975687

## Background

Chimeric antigen receptor-modified T cells (CAR T cells) directed against CD19 have shown promise as a novel therapy for hematological malignancies; complete remission (CR) has been achieved in as many as 70–90% of cases of relapsed/refractory acute lymphoblastic leukemia (R/R B-ALL) [1–5]. Unfortunately, 40–50% of patients responding to this therapy relapse within 1 year, and nearly half of these relapses included CD19-positive leukemic cells [2–5], while many efforts have been made to improve the design and production of the CAR [6–8]. Recent studies indicate that relapse may be due to mutations in CD19 that destroy the cognate epitope recognized by the anti-CD19 single-chain variable fragment (scFv), such as FMC63 clone, thus rendering the tumor cells invisible to CD19 CAR T cells [9, 10]. In cases such as these, an exploration of CD19 CAR T with scFvs capable of binding to different CD19 epitopes may provide alternative choice for patients with mutations in CD19. Results from our previous study revealed that CD19 CAR T cells constructed in our laboratory that were derived from the new HI19α clone (HI19α-4-1BB-ζ CAR T, or CNCT19) were highly effective when evaluated in preclinical models [11]. Here, we showed that the scFv generated by the HI19α clone detects a different binding epitopes on the CD19 extracellular domain (ECD) than that detected by FMC63. And we present a single-arm, open-label, non-randomized, prospective pilot study that was conducted to evaluate the safety and efficacy of CNCT19 cells in both children and adult patients with R/R B-ALL.

Furthermore, it is also critical to recognize that despite the impressive initial CR rates associated with current CD19 CAR T therapies for B-ALL, long-term follow-up has revealed that a significant proportion of patients relapse after treatment. As such, more efforts are needed to identify biomarkers that can predict the long-term response to CAR T cell therapy. Retrospective analyses from clinical trials indicate that inadequate clinical responses to CAR T cell therapy might also be associated with an insufficient expansion and persistence of functional CAR T cells in vivo [3, 12]. Preclinical human tumor xenograft models have been used to examine the potency of genetically modified T cells derived from different subsets that are isolated prior to transduction [13–15]. These results revealed that CAR T cells generated from less differentiated T cell subsets exhibited more proliferative potential and anti-tumor activity than did those derived from differentiated cell subsets [16–18]. However, it is not yet clear whether the expansion kinetics of different T cell subsets are associated with differential responses observed with respect to long-term patient responses to therapy. Likewise, the anti-tumor capabilities of some T cell subsets, such as CD8^+^ central memory T cells (T_CM_), remain controversial and have not been subjected to adequate study [13, 19, 20]. Considering the fact that the CAR T cells administered to patients may interact with components of the endogenous immune system in vivo [21], it will be important to resolve issues associated with expansion kinetics and to understand the functional capabilities of different T cell subsets during the course of treatment in order to identify potential biomarkers that will predict long-term responses.

In this research, we analyzed the kinetics of various T cell subsets collected from the peripheral blood of patients after CAR T cell infusion. Our findings suggest that the percentage of CD8^+^ naïve T cells (T_N_) represents a potential biomarker that might be developed as a means to predict the long-term prognosis of patients undergoing CAR T cell therapy.

## Methods

### Human CD19 structure and antibody homology modeling

The crystal structure of human CD19 (hCD19) is publicly available (PDB ID: 6AL5). However, this structure is not the ECD of CD19. The CD19 3D structure was remodeling based on 6AL5 by using the “Create homology Models” program in Discovery Studio [22]. All models were ranked by PDF total energy. Choose the structure with the lowest PDF total energy for further modeling. This rough structure was analyzed using Ramachandran plot and “Profiles-3D Verify” program and then optimized using “Loop Refinement”. The scFv homology models of HI19α and FMC63 were generated based on their amino acid sequences, using the “Create homology Models” module in Discovery Studio [8]. The sequence of each antibody was searched in the structure database of PDB to find its best templates. All BLAST hits were ranked by *E* value. Choose 5 templates with high resolution (< 2.8 Å) for further modeling. One hundred models were constructed for each antibody. The final model was chosen based on its PDF total energy, Ramachandran plot and Profile-3D verify result.

### Antibody-antigen docking

The binding mode between hCD19 and HI19α (or FMC63) was performed by rigid body docking program ZDOCK and integrated in Discovery Studio. Keeping the position of antibody fixed as a receptor, the hCD19 model was rotated around the receptor in a rigid-body manner to search possible binding poses. Fifty-four thousand poses were generated after each ZDOCK and ranked by ZDOCK score. Only those poses with high ZDOCK score (> 12) were selected for further optimization. Furthermore, by knowing that CDR loops on antibodies are the roughly binding sites, additional filtering process was performed to narrow down the scope of refinement. All qualified poses were typed with the CHARMm Polar H forcefield and refined using B RDOCK program. Choose the final binding poses based on RDOCK scores and protein binding interface.

### The antibody competition experiment

1.3 × 10^5^ Nalm-6 cells were stained with 0.0112, 0.0168, 0.0336, 0.0420, or 0.0672 pM FMC63 for 30 min at 4 °C, respectively, washed twice, and then stained with 0.02 μg/μl anti-mouse IgG (H+L), F(ab’)_2_ Fragment for 30 min at 4 °C. 1.3 × 10^5^ Nalm-6 cells were stained with 0.0068, 0.0136, 0.017, 0.034, or 0.068 pM HI19α-PE antibody for 30 min at 4 °C, respectively. The minimum binding concentration of antibody was determined. Then, 1.3 × 10^5^ Nalm-6 cells were stained with 0.0420 pM FMC63 for 30 min at 4 °C, washed twice, stained with 0.02 μg/μl F(ab’)_2_-Alexa Fluor 647 for 30 min at 4 °C, and washed twice. And then stained with 0.017 pM HI19α-PE for 30 min at 4 °C. After staining, all the cells were washed twice and suspended in PBS for flow cytometry analysis.

### Clinical trial design and patients

We conducted an open-label, single-center, and single-arm pilot study to evaluate the safety and efficacy of CD19 CAR T cells derived from a new clone HI19α into patients with CD19^+^ R/R B-ALL at the Institute of Hematology & Blood Diseases Hospital, Chinese Academy of Medical Sciences & Peking Union Medical College. The patients were eligible if they were CD19^+^ R/R B-ALL who fail to achieve remission after first or more salvage treatment. Philadelphia chromosome (Ph)-positive B-ALL patients must have failed treatment with at least 1 second-generation tyrosine kinase inhibitor (ponatinib is unavailable in China) or with T315I mutation. Patients were excluded if they had an active central nervous system leukemia (CNSL) and insufficient performance status (Eastern Cooperative Oncology Group performance score ≥ 3) or organ function. The primary and secondary objectives are the safety and efficacy of CNCT19 in childhood and adult patients with R/R B-ALL, respectively.

### CD19 CAR T construction and generation

The CD19 CAR T cells were produced by Juventas Cell Therapy Ltd (Product ID: CNCT19). The scFv sequence specific for CD19 was derived from clone HI19α, which was established at our hospital. Anti-CD19 scFv combined to human 4-1BB/CD3-ζ costimulatory signaling components were cloned into a lentiviral backbone to obtain lentiviral vectors pCDH-HI19α-4-1BB/CD3ζ-CAR. The lentivirus packaging and CAR T cell transduction and expansion were performed as described [11, 23]. In addition, CD19 CAR T cultures were checked twice for possible contamination of fungus, bacteria, mycoplasma, chlamydia, and endotoxin and subsequently stored at − 80 °C until use.

### Clinical protocol design and assessment of toxic effects

Prior to CAR T cell infusion, all patients underwent FAC lymphodepletion chemotherapy that included cyclophosphamide (350 mg/m^2^/day, days -4 and -2), fludarabine (30 mg/m^2^/day, days -4, -3, and -2), and cytarabine (100 mg/m^2^/day, days -4, -3, -2, and -1). No other treatments were administered, prescribed, or modified as part of this study. Total T cells were administered at a dosage of 5 × 10^6^ cells per kilogram (kg) of body weight. The median efficiency of the final CAR T cell transduction was 0.41; as such, the median CAR T cell concentration reached 2.02 × 10^6^/kg body weight (range 0.75 × 10^6^–4.08 × 10^6^ cells/kg body weight) in this study. The patients underwent bone marrow (BM) examination on day 14 and day 28 to determine the remission status. CR rate and relapse were evaluated in accordance with the National Comprehensive Cancer Network (NCCN) guidelines, version 1.2016. Minimal residual disease (MRD) negative response is defined as less than 10^− 4^. BM blasts are determined by flow cytometry assays. And no target amplification with a minimum sensitivity of 10^− 4^ by quantitative real-time polymerase chain reaction in Ph^+^ B-ALL. The presence of CAR T cells and phenotype of T cells was detected and quantified using multiparameter flow cytometry in the peripheral blood from all patients. Surface marker staining was performed to assess the corresponding subpopulation markers on regulatory T cells (Tregs, CD4^+^ CD25^+^ CD127^−^), naïve T cells (T_N_, CD45RA^+^ CCR7^+^), central memory T cells (T_CM_, CD45RA^−^ CCR7^+^), effector memory T cells (T_EM_, CD45RA^−^ CCR7^−^), and effector T cells (T_E_, CD45RA^+^ CCR7^−^). The antibodies were listed in Supplementary Table 1. The cytokine release syndrome (CRS) was graded and managed according to Lee’s grading system for CRS [24]. Grade of adverse event was evaluated over the whole duration of the treatment, an expected average of 24 months using the National Cancer Institute Common Terminology Criteria for Adverse Events (NCI-CTCAE) version 4.03 (https://ctep.cancer.gov/).

### Statistical analysis

Overall survival (OS) was calculated from the date of enrolled to the date of death or last follow-up. Relapse-free survival (RFS) was calculated from the date of CR/CRi to the date of relapse or death, or the last follow-up. The independent *t* test and Mann-Whitney non-parametric test was used for the analysis of continuous variables. The chi-square test was used for categorical data. The probabilities of OS and RFS were estimated by means of the Kaplan-Meier. Both univariate and multivariable Cox regression analyses were applied to determine whether the T cell percentage contributed to the long-term response. All *p* values represented were two-sided and considered statistically significant when *p* < 0.05. Statistical analyses were performed with SPSS 19.0 software.

## Results

### FMC63 scFv and HI19α scFv detect distinct epitopes within the extracellular domain of CD19

**Fig. 1:**
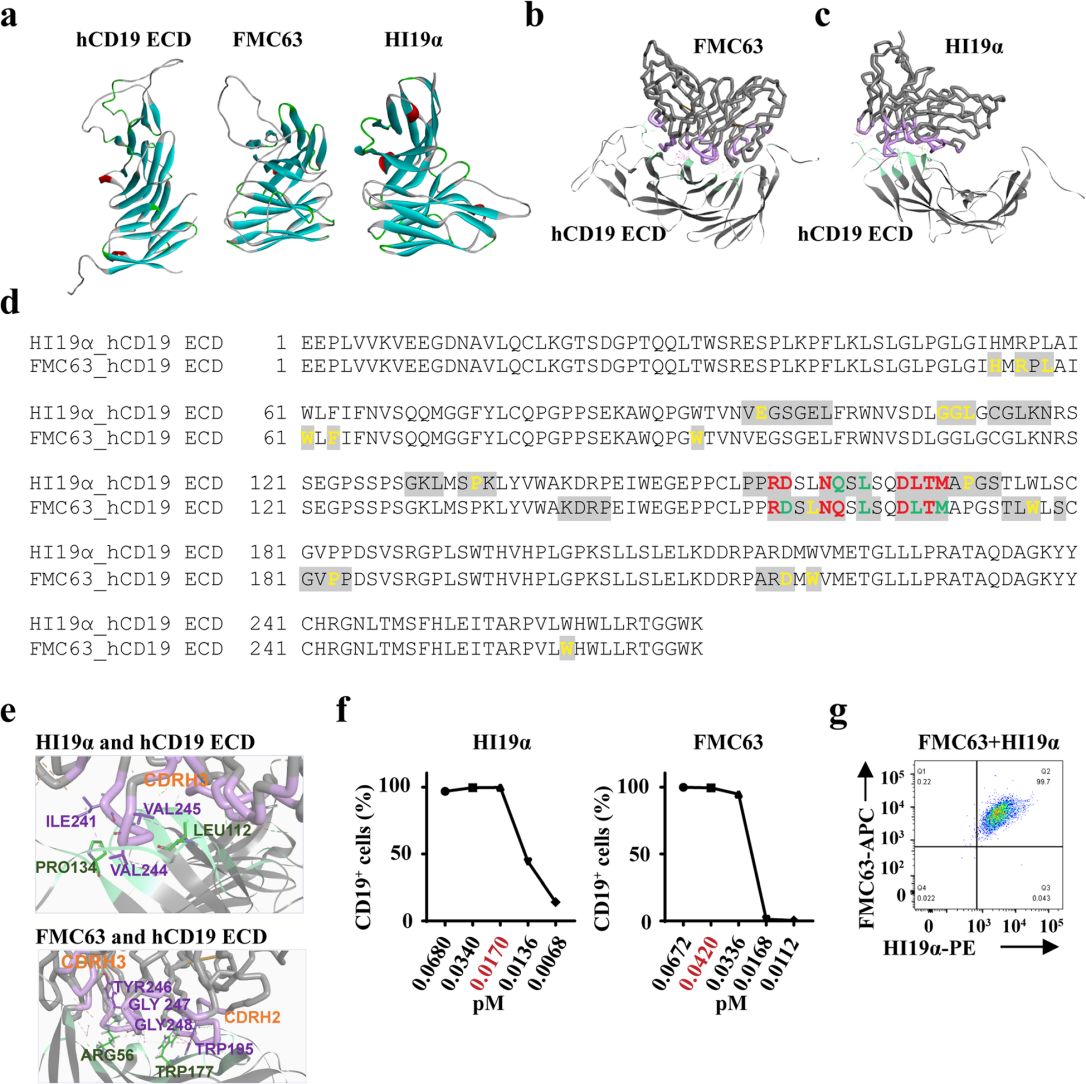
FMC63 and HI19α had different binding epitopes on CD19. **a** Homology models of hCD19 ECD (left), FMC63 scFv (middle), and HI19α scFv (right). **b** Docking mode of FMC63 and hCD19 ECD. Green, hCD19 ECD epitopes; purple, FMC63 epitopes. **c** Docking mode of HI19α and hCD19 ECD. Green, hCD19 ECD epitopes; purple, HI19α epitopes. **d** Sequence of hCD19 ECD. Gray background, predicted binding amino acid residues on hCD19 ECD with scFvs. Upper panel, interaction with HI19α; lower panel, interaction with FMC63. Red color, shared antigen epitopes; yellow color, key epitopes; green color, both shared antigen epitopes and key epitopes. **e** Partial interaction modes showed the non-bond interaction between scFvs and hCD19 ECD. Upper panel, HI19α scFv and hCD19 ECD; lower panel, FMC63 scFv and hCD19 ECD. Green, hCD19 ECD epitopes; purple, scFvs epitopes. **f** Flow cytometry analysis of the proportion of CD19^+^ Nalm-6 cells stained with indicated concentrations of the antibody. Left panel, HI19α antibody; right panel, FMC63 antibody. **g** Representative flow cytometry analysis showing the proportion of CD19^+^ cells on Nalm-6 cells stained with 0.017 pM HI19α and 0.042 pM FMC63

Currently, the scFv derived from hybridoma clone FMC63 is typically incorporated into targeted CD19 CAR T cell products. Here, we generated novel CD19 CAR T cells with the scFv derived from novel hybridoma HI19α. To determine whether these two different scFvs detect distinct epitopes on hCD19, the structure of the hCD19 ECD, the FMC63 scFv, and the HI19α scFv were evaluated by molecular modeling (Fig. 1a). The binding interactions between hCD19 and HI19α and hCD19 and FMC63 were evaluated by the rigid body docking program ZDOCK; the findings were integrated with Discovery Studio (Fig. 1b, c). The amino acids of hCD19 ECD predicted to be involved in binding interactions are shown in Fig. 1d (gray background). Our initial results revealed that the hCD19 epitopes detected by the two scFvs were not identical. To identify the key antigen epitopes, alanine scanning mutagenesis was performed at the binding interface for each complex (Fig. 1d). After the introduction of each mutation, an increase in binding energy by > 0.5 was used to identify the critical amino acid residues (Fig. 1d; Supplementary Tables 2 and 3). The results revealed an overlap between the critical amino acid residues involved in the interactions between the HI19α scFv and the FMC63 scFv with the hCD19 ECD (Fig. 1d). The interaction between each of the scFvs and the hCD19 ECD involved non-covalent bonds (Supplementary Tables 4 and 5); partial interaction modes are as shown in Fig. 1e. To verify the findings from the docking model, minimum binding concentrations of each of the two scFvs were determined in an experiment targeting Nalm-6 cells; nearly all of these cells express CD19 (Fig. 1f). The minimum binding concentrations of the FMC63 and HI19α scFvs were 0.042 and 0.017 pM, respectively. Antibody competition experiments were also performed; the results revealed that Nalm-6 cells could be detected simultaneously with both antibodies (Fig. 1g). These results provided further confirmation that the FMC63 and HI19α scFvs interacted with distinct epitopes within the hCD19 ECD.

### Characteristics and response rates of patients diagnosed with R/R B-ALL

**Fig. 2:**
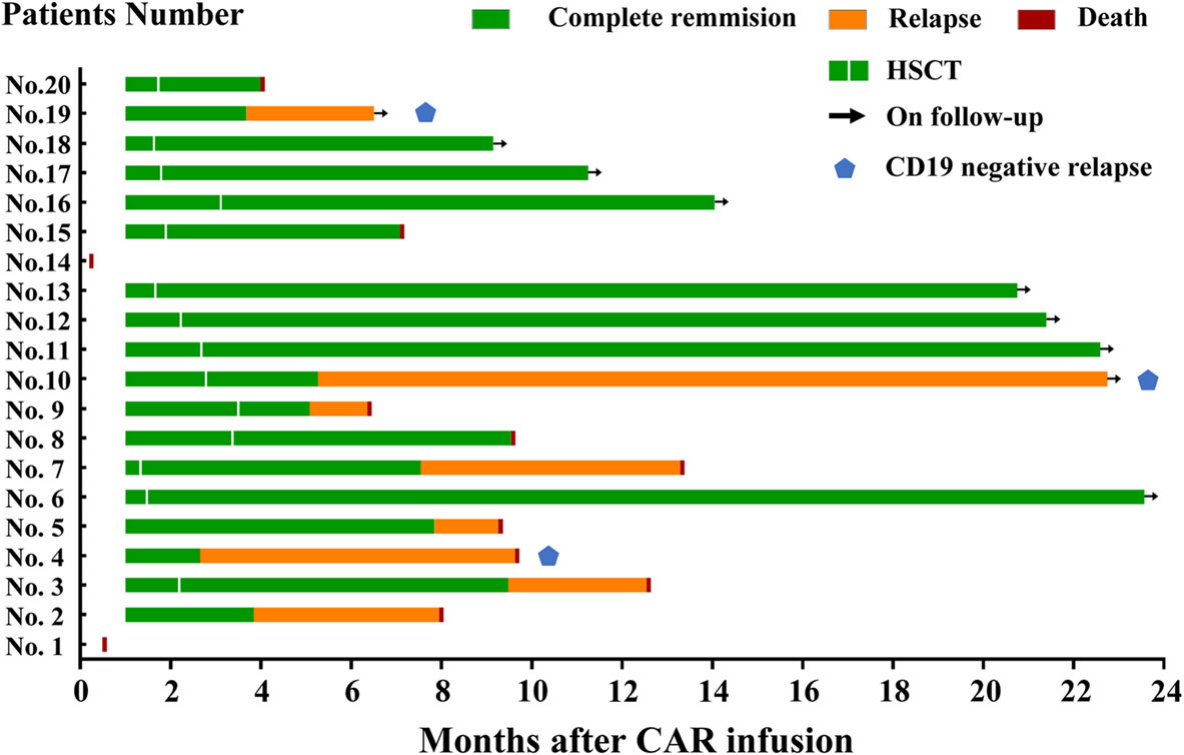
Responses of all 20 R/R B-ALL patients to CNCT19 CAR T cell therapy. In this single-center, open-label, prospective clinical trial, a total of 20 patients diagnosed with resistant or refractory CD19^+^ B cell acute lymphoblastic leukemia received an infusion of CNCT19 CAR T cells. Responses over time of each patient are presented as a swimmer plot

To assess the safety and feasibility of CNCT19 cell therapy in patients with R/R B-ALL, 24 childhood and adult were screened from January 6, 2017, through August 12, 2019; two patients were not enrolled in the study for the reasons listed in Supplementary Fig. 1. A total of 22 patients undergo an attempt at CAR T production; two of whom did not have cells infused (one owing to drawing consent, one because of production failure). As such, a total of 20 patients received an infusion of CAR T cells. At enrollment, these patients were at the median age of 18 years old (range, 3–52 years old). The median percentage of abnormal B cells in BM specimens from these 20 patients prior to lymphodepletion chemotherapy was 35.5% (range, 3–96%); two patients had both BM-associated and extramedullary disease (Table 1). Complex chromosomal aberrations, including high-risk fusions and mutations including *TP53* and *IKZF1* deletions, were identified in 8 (40%) patients. The rate of CR/CRi achieved by the 20 patients who received CAR T cell infusion was 90% at day 28 (Fig. 2). In a further intention-to-treat analysis of the full enrolled 22 patients, the CR/CRi rate at day 28 was 82%. After infusion of CNCT19 cells, two patients died within 28 days due to CAR T cell-related encephalopathy syndrome (CRES); the 18 surviving patients achieved MRD-negative remission (100%) within 3 months. The two patients with extramedullary disease in the lumbar spine or skin also achieved CR; this was confirmed in BM aspirates, skin biopsies, and computed tomography scans. Taken together, these results demonstrate that CD19 CAR T cell therapy with CNCT19 cells results in a rapid induction of CR that is effective for patients diagnosed with R/R B-ALL involving both BM and in extramedullary sites. This therapeutic option is effective in patients with complex chromosomal aberrations, including high-risk gene fusions and mutations.

**Table 1:** Clinical characteristics of 20 R/R B-ALL patients

Patient number	Age	Gender	ECOG	Leukemia type and genetic abnormalities	Disease status	BM blasts burden	Prior lines of treatment	EMD status
1	31	Female	1	Ph-ALL	Refractory	56.0%	2	No
2	23	Female	1	Ph+ALL*(T315I mutation)	Relapse	38.6%	2	No
3	50	Male	0	Ph-ALL	Refractory	3.0%	2	No
4	18	Male	0	Ph-ALL	Relapse	30.5%	2	No
5	44	Male	0	Ph-ALL	Refractory	24.5%	2	No
6	16	Male	0	Ph-ALL (IKZF1 deletion, NRAS mutation)	Refractory	26.0%	3	No
7	10	Male	0	Ph-ALL (E2A-PBX1 rearrangement)	Relapse	4.5%	2	No
8	16	Male	0	Ph+ALL (T315I mutation)	Relapse	83.5%	2	No
9	24	Male	1	Ph-ALL	Relapse	40.5%	2	No
10	9	Male	0	Ph-ALL (IgH rearrangements)	Refractory	40.5%	2	No
11	7	Male	0	Ph-ALL	Relapse	7.5%	2	No
12	9	Female	0	Ph-ALL (ETV6-RUNX1 rearrangement)	Relapse	10.0%	2	No
13	19	Male	0	Ph-like ALL (EBF1-PDGFRB rearrangement, IKZF1 deletion)	Relapse	75.0%	2	Yes
14	14	Female	0	Ph-ALL	Refractory	32.5%	3	No
15	18	Male	0	Ph-like ALL (PAX5-JAK2 rearrangement, TP53 deletion)	Relapse	73.5%	1	No
16	43	Female	1	Ph-ALL	Refractory	60.5%	2	No
17	11	Female	0	Ph-ALL	Refractory	5.0%	2	No
18	3	Female	0	Ph-ALL (TP53 mutation, IKZF1 deletion)	Refractory	6.0%	5	No
19	52	Female	0	Ph-ALL	Refractory	96.0%	2	No
20	40	Male	0	Ph-ALL (IKZF1 deletion)	Refractory	64.5%	2	Yes

*EMD* extramedullary disease, *Ph* Philadelphia chromosome

### Long-term survival

**Fig. 3:**
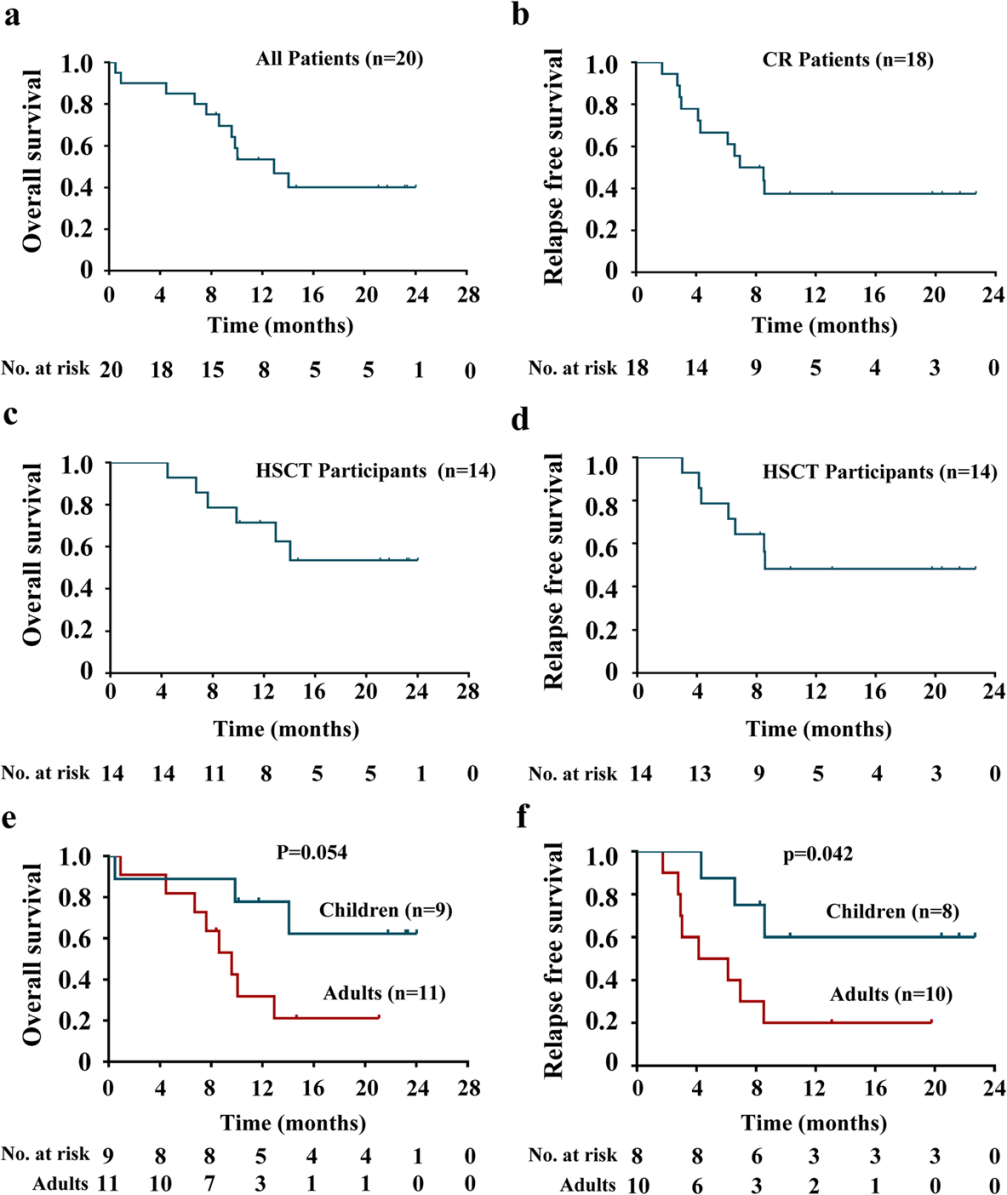
Long-term survival. **a**–**b** Overall survival (**a**) and relapse-free survival (**b**) of the 20 R/R B-ALL patients treated with the CNCT19 CAR T cell infusion. Overall survival (**c**) and relapse-free survival (**d**) of patients who underwent hematopoietic stem cell transplantation. Overall survival (**e**) or relapse-free survival (**f**) of both pediatric and adult patients

A total of 11 patients did not survive to the median follow-up time of 10.09 months (range, 0.49–24.02 months); as noted earlier, this included the two patients who developed CRES within 28 days of the infusion, as well as six who experienced relapse and disease progression, and three who experienced complications associated with hematopoietic stem cell transplantation (HSCT). The median OS reached 12.91 months (95% confidence interval [CI], 7.74–18.08 months; Fig. 3a) for the patients who received CAR T cell infusion. Among the 18 patients who achieved CR, eight patients eventually relapsed; five of these relapses involved CD19-positive cells and three were CD19-negative. There were no central nervous system-associated relapses in this patient cohort. The median RFS for the entire patient cohort was 6.93 months (95% CI, 3.13–10.73 months; Fig. 3b). Fourteen patients underwent allogeneic HSCT after treatment with the CD19 CAR T cell infusion. The median time from the CAR T cell infusion to HSCT was 61 days (range, 40–106 days). The median OS for patients who underwent allogeneic HSCT was not reached. One-year OS rate and RFS rate of patients who bridge to allogeneic HSCT after CAR T cell therapy was 62.2% (95% CI, 31.2–82.4%) and 49.2% (95% CI, 21.9–71.7%), respectively (Fig. 3c, d). Childhood (age < 18 years old) experienced longer OS and RFS than did the adult patients (*p* = 0.054 and 0.042, respectively), although these differences did not reach statistical significance on OS (Fig. 3e, f).

### T cell phenotypes and correlation with long-term survival

**Fig. 4:**
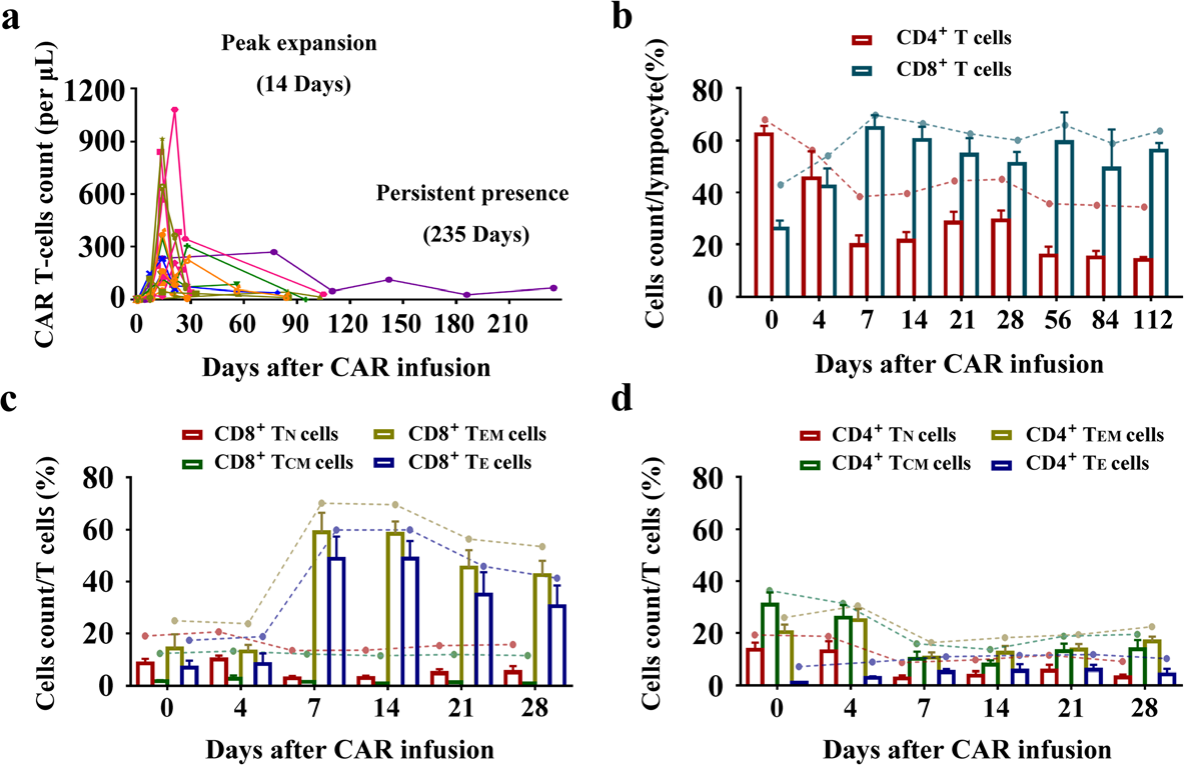
The expansion kinetics of CAR T cells and different T cell subsets after CD19 CAR T cell infusion. **a** The expansion and persistence of CNCT19 CAR T cells after infusion. **b** The expansion and percentage of CD4^+^ or CD8^+^ T cells in peripheral blood after CAR infusion. **c**–**d** The phenotype of CD8^+^ T cells (**c**) or CD4^+^ T cells (**d**) in peripheral blood after CNCT19 CAR T cell infusion (T_N_, naïve T cells; T_CM_, central memory T cells; T_EM_, effective memory T cells; and T_E_, effector T cells)

**Fig. 5:**
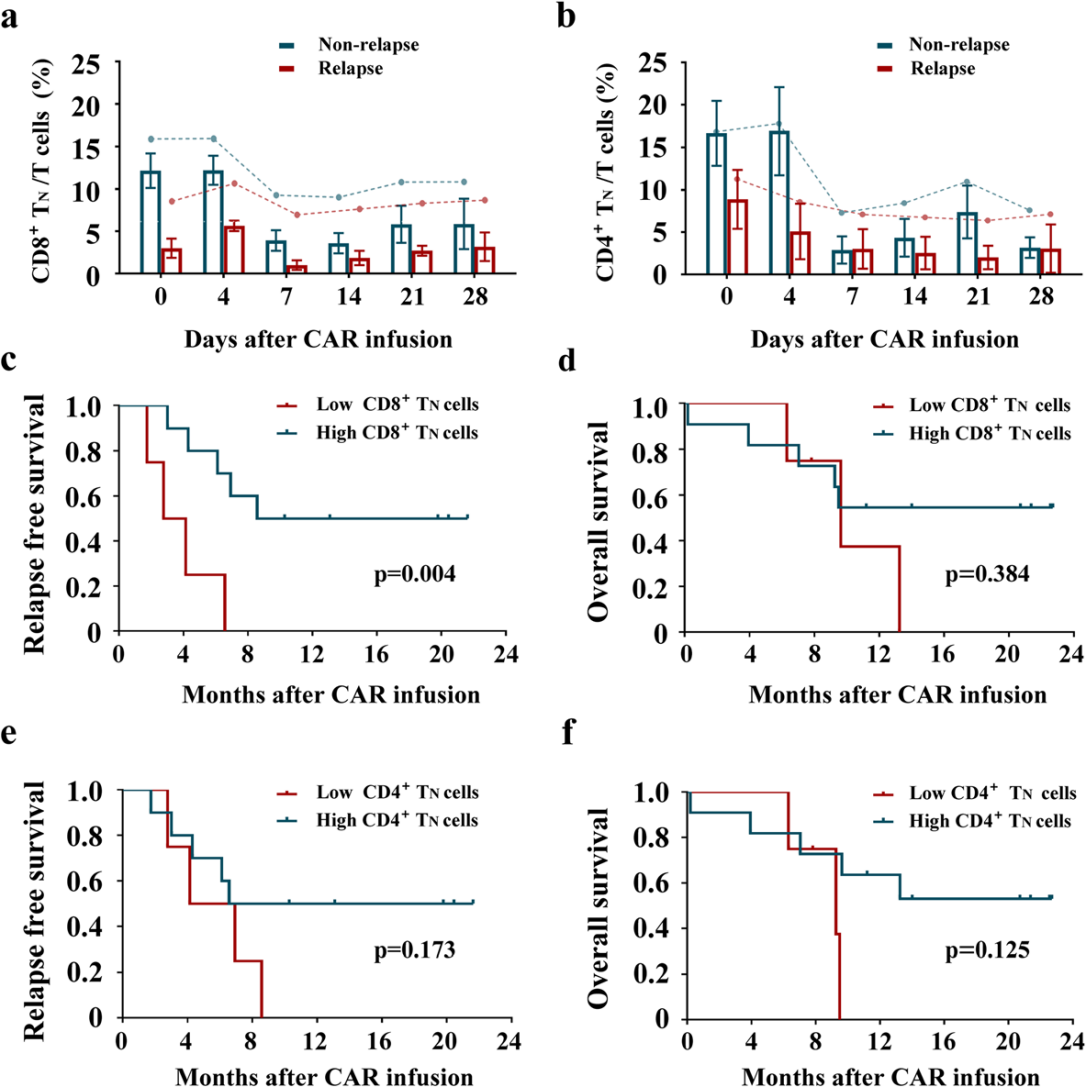
The correlation between T_N_ cell percentage and long-term survival. **a**, **b** Comparison of CD8^+^ T_N_ cells (**a**) or CD4^+^ T_N_ cell (**b**) percentage in peripheral blood at different time points after CAR T cell infusion between long-term response and relapsed patients (T_N_, naïve T cells). CD8^+^ or CD4^+^ T_N_ cells percentage were ranked and divided into four quartiles, from lowest (Q1) to highest (Q4). **c**, **d** Relapse-free survival (**c**) or overall survival (**d**) difference between patients with low CD8^+^ T_N_ cells percentage (Q1) and those with high CD8^+^ T_N_ cells percentage (Q2–4). **e**, **f** Relapse-free survival (**e**) or overall survival (**f**) difference between patients with low CD4^+^ T_N_ cells percentage (Q1) and high CD4^+^ T_N_ cells percentage (Q2–4)

In an effort to identify factors associated with the long-term response to therapy, we assessed the CD19 CAR T cell percentages and T cell phenotypes in peripheral blood by flow cytometry at serial time points after the CAR T cell infusion. We found that CAR T cells can be detected in the blood for up to 235 days after infusion (Fig. 4a); the greatest expansion of these cells was detected at day 14 among the 20 patients who received CAR T cell infusion as part of this trial. After CD19 CAR T cell infusion, we detected a gradual decrease in the fraction of CD4^+^ T cells accompanied by a significant increase in the percentage of CD8^+^ T cells (Fig. 4b). Further evaluation of these T cell subsets revealed gradual reductions in the percentages of both CD8^+^ and CD4^+^ T_N_ (naïve) and T_CM_ (central memory) cells after CD19 CAR T cell infusion. Percentages of both CD8^+^ T_EM_ (effector memory) and T_E_ (effector) cells increased significantly during the first 2 weeks after infusion; these percentages then decreased gradually, while the percentages of CD4^+^ T_EM_ and T_E_ cells were maintained at relatively stable levels (Fig. 4c, d). These results suggest that the T_N_ and T_CM_ cells may undergo gradual differentiation into T_EM_ and T_E_ cells that are the critical mediators of CD19^+^ cell death. Interestingly, we identified a significantly reduced percentage of CD8^+^ T_N_ cells shortly after the CD19 CAR T cell infusion in patients who eventually relapsed from B-ALL compared to that in patients identified as long-term responders (*p* = 0.006; Fig. 5a). Similarly, the percentage of CD4^+^ T_N_ cells detected shortly after the CD19 CAR T cell infusion was lower in patients who ultimately experienced relapse, although these differences did not reach statistical significance (Fig. 5b). No differences in the percentages of T_CM_, T_EM_, or T_E_ cells were detected at any of the time points evaluated (Supplementary Fig. 2). To explore the relationship between the percentage of T_N_ cells and the likelihood of relapse, we ranked and divided the CD8^+^ T_N_ cells percentages detected in each patient shortly after CD19 CAR T cell infusion into four quartiles, from lowest (Q1) to highest (Q4). The patients with CD8^+^ T_N_ cell percentages in Q1 experienced shorter periods of RFS (*p* = 0.004; Fig. 5c), although no association with OS was detected (*p* = 0.384; Fig. 5d). To confirm the outcomes and adjust for potential confounding factors, we constructed multivariate Cox models to test the proportional hazards assumption as well as the interaction terms with covariates. The variables included in the Cox models were those associated with *p* values < 0.1 in univariate analyses (Supplementary Table 6). The multivariate analysis indicated that the percentage of CD8^+^ T_N_ cells emerged as an independent negative prognostic factor with respect to RFS (*p* = 0.012, 95% CI, 0.017–0.601; Supplementary Table 7). The percentage of CD4^+^ T_N_ cells detected shortly after CD19 CAR T cell infusion was not found to be associated with a shorter period of RFS or OS (*p* = 0.173 and 0.125, respectively; Fig. 5e, f). Although the fraction of Tregs also increased in response to CD19 CAR T cell infusion (Supplementary Fig. 3a), no changes in the percentages of CD3^+^CD56^+^CD16^+^NK-like T cells or NK cells were observed (Supplementary Fig. 3b); this is intriguing, given the recent reports focused on NK cells and their unexpected regulatory role in outcomes associated with BM transplantation.

The fraction of CD19^+^ B cells was evaluated as a means to track potential relapse of B-ALL. After CD19 CAR T cell infusion, CD19^+^ B cells were eliminated within 3 months in 18 of the 20 patients. Interestingly, although relapses were detected in most patients within 5–20 days after the appearance of circulating CD19^+^ B cells, relapse in two patients was delayed to > 130 days after CD19^+^ B cell detection (Supplementary Fig. 4a, b); of note, relapse occurred despite persistence of CD19 CAR T cells for > 180 days in peripheral blood or BM in both of these patients.

### Toxicities associated with CNCT19 CAR T cell therapy

The safety analysis set included all 20 patients who received an infusion of CNCT19 cells. The most significant treatment-related toxicities were CRS and CRES. Overall, the CRS was detected in 95% (19/20) patients and was severe (grade ≥ 3) in 9/20 (45%). The median onset of CRS was 4 days after the CAR T cell infusion; this complication lasted for an average duration of 8 days. The clinical signs and symptoms of CRS include fever, nausea, vomiting, hypotension, hypoxemia, and musculoskeletal pain. Hypofibrinogenemia, hyperbilirubinemia, and elevated levels of liver aminotransferases were the most common severe toxicities detected in association with specific organ systems, including six patients (30%) with grade 3 hypofibrinogenemia that resolved within 2 months after the CAR T cell infusion. One patient (5%) experienced grade 3 hyperbilirubinemia, and another patient (5%) developed grade 3 elevations in liver aminotransferases. No severe abnormalities associated with renal or cardiovascular functions were observed (Table 2). CRES was diagnosed in 13 patients (65%); eight of these patients experienced grade ≥ 3 CRES, while two patients reached grade 5. The most common neurologic events associated with CRES were headache (20%), confusion (5%), delirium (20%), agitation (20%), and seizures (20%). The signs and symptoms associated with CRES were relieved by dexamethasone treatment. Furthermore, and as anticipated, all patients responded to CAR T cell infusion with reversible hematologic toxicity; four patients developed grade 3 infections although none experienced severe bleeding.

**Table 2:** Summary of adverse events in the 20 patients receiving CAR T cell infusion

TEAEs (***N*** = 20)	Any grade (%)	Grades 3–4 (%)	Grade 5 (%)
Cytokine release syndrome	95	45	0
Neurologic event	65	30	10
Hematologic event			
Thrombocytopenia	95	90	0
Anemia	100	70	0
Neutropenia	100	100	0
Non-hematologic event			
Diarrhea	50	0	0
Abdominal pain	15	0	0
Insomnia	5	0	0
Headache or dizziness	20	0	0
Muscle or bone pain	25	0	0
Capillary leak syndromes	30	0	0
Infection	20	20	0
Hypotension	25	5	0
Hypoxemia	30	5	0
Elevated aminotransferases	60	5	0
Elevated blood bilirubin	60	5	0
Elevated creatinine	0	0	0
Cardiovascular disorders	0	0	0
Hyponatremia	20	0	0
Hypokalemia	55	0	0
Hypocalcemia	65	0	0
Hypoalbuminemia	50	0	0
Hypofibrinogenemia	60	30	0
Prolonged activated partial thromboplastin time	15	0	0

## Discussion

A growing body of evidence has confirmed the high clinical efficacy of CAR T cells that target CD19, notably for the treatment of B cell-associated hematological malignancies [1, 25–29]. Unfortunately, patients treated with CAR T cells remain prone to relapse; some of them may be mediated by mutations that develop in the tumor cell epitope which impair binding interactions with specific CAR T cell scFv [9, 10]. Recent clinical trials seeking to improve the response to CAR T cell therapy have included numerous changes, ranging from modification of the costimulatory domain to varying the treatment regimen; however, the CAR T scFv used in these trials remain primarily those derived from hybridoma clones FMC63 or SJ25C1. Here, we report the safety and efficacy of infusions performed with CD19 CAR T cells derived from a new hybridoma clone HI19α with scFvs that target distinct epitopes of hCD19 antigen. Data from our study suggested that 90% of patients achieved CR/CRi within 28 days after the CAR T cell infusion, which accounted for 82% of the fully enrolled 22 patients in an intention-to-treat analysis; these values are comparable to those obtained in the pivotal ELIANA study, which included an overall remission rate of 81% in patients receiving CAR T cell infusion and 66% when accounting for all fully enrolled patients [30]. The median OS and RFS in response to CNCT19 cells infusion were similar to findings published previously, including those observed in response to 19–28ζ CAR T cells generated at the Memorial Sloan Kettering Cancer Center (MSKCC), New York, USA (i.e., median OS of 12.9 vs. 12.9 months and median RFS of 6.9 vs. 6.1 months) [12]. Our results suggest that the CD19 CAR T cells derived from the HI19α clone are also capable of promoting high anti-leukemic efficacy and may be able to address issues associated with poor prognosis in R/R B-ALL patients. Nonetheless, our results need to be interpreted with caution because 14 of the 20 patients in our study underwent subsequent consolidation with HSCT; this fraction was much higher than reported among the patients in the MSKCC’s trial (17/53) and may play a significant role in maintaining the long-term response to treatment [30]. As such, the duration of the response associated specifically with CNCT19-based T cell therapy requires further investigation. In addition, while CARs with alternative targets and humanized CD19-CAR have been reported to be attractive in addressing the CD19^+^ relapse after CD19-CAR T cell therapy [31, 32], whether CNCT19 could provide an alternative choice for those patients, especially in patients with CD19^+^ relapse from CAR T therapy based on the clone FMC63, is still worthy of research. And before exploring that issue, humanized modification of CNCT19 may be necessary considering the potential immune-mediated rejection on the murine origin domains of CAR T cells [29, 33].

Although CNCT19 cells are therapeutically effective in patients with R/R B-ALL, the associated toxicities, which occurred in all studies featuring infusions of CAR T cells, remain a significant concern. In our study, high-grade CRS developed in 45% of patients after CNCT19 cells infusion; this result is similar to that observed in the ELIANA study, in which severe CRS was reported in 47% of the children and young adults with R/R B-ALL who were treated with tisagenlecleucel [30]. Severe CRS associated with CNCT19 T cells infusion was observed predominantly among patients with high tumor burden; severe CRS developed in 85.7% (6/7) of patients with 50% or more BM blasts, but only in 23% (3/13) of the patients with fewer than 50% blasts in the BM. This is consistent with results reported by Maloney et al. [29], which discussed the role of tumor burden and the associated risks of severe CRS and neurotoxicity. These results highlight the need to decrease the tumor burden prior to the CAR T cell infusion. Of note, CNCT19 cells seem to be associated with relatively high rate of severe neurotoxicity; 40% of the patients receiving CNCT19 cells infusion experienced severe neurotoxic complications, compared with 13% in the ELIANA study. The mechanisms underlying the observed neurotoxic responses to CAR T cell therapy remain unclear. According to previously published data, severe CAR T cell-associated neurologic events were reported in ~ 5–21% of children and young adults [2, 26, 30] and in ~ 42–50% of adult patients with R/R B-ALL [12, 29]. Severe neurotoxicity appears to be more commonplace among adult patients, although the heterogeneity with respect to the specific CAR T cell preparations as well as the characteristics of the patients enrolled in each study do not permit direct comparisons to be performed. The analyses from MSKCC also suggested a possible association between neurotoxicity with the age of the patients, although these results did not reach significance [12]. In light of this, the relatively older age of the patients enrolled in our trial may provide a partial explanation for the higher incidence of neurotoxicity observed here than ELIANA study. Furthermore, differences with respect to lymphodepletion strategies and expansion kinetics associated with CAR T cells may also influence their overall safety profiles. To the best of our knowledge, we describe here the first clinical trial that features an FAC regimen (fludarabine, cytarabine, cyclophosphamide) for lymphodepletion prior to CAR T cell infusion. While more aggressive lymphodepletion may help promoting the initial expansion and efficacy of CAR T cells by diminishing the regulatory mechanisms and increasing the availability of homeostatic cytokines, we cannot rule out the possibility that rapid and extensive expansion of the CAR T cell population may result in an accelerated release of proinflammatory cytokines and an increased incidence of severe adverse events [34–36]. Meanwhile, aggressive lymphodepletion may result in broad impairment of normal tissues and endothelial activation, which may also ultimately trigger a higher incidence of severe toxicity [36, 37]. As such, additional studies will be needed to explore the impact of various lymphodepletion regimens with respect to the efficacy vs. the toxicity associated with adoptive T cell therapy. It will be critical to develop one or more lymphodepletion strategies that optimally balance the risk and benefit for the patients. And more importantly, several safety strategies of CAR T cells, including suicide genes, on-switch CAR, combinatorial target-antigen recognition, and bispecific T cell engager have been developed to reduce the side effects, which may provide a great opportunity toward improving the safety of CNCT19 [38].

Recent studies that have explored various other factors associated with relapse after HSCT have focused on the differential expansion of T cell subsets. It was initially presumed that a comparatively high level of T_E_ cells, which demonstrate immediate cytolytic capabilities, would correlate with long-term anti-tumor activity. However, emerging data from preclinical studies suggest that terminally differentiated T cell subsets display impaired anti-tumor responses in vivo [39, 40] and that a high percentage of one or more subsets of less differentiated T cells may contribute to the prolonged in vivo persistence of functional CAR T cells [14, 19, 41]. Further research has revealed that human CD8^+^ T cells derived from less differentiated subsets, such as T_N_ cells [42, 43], exhibit superior traits with respect to adoptive immunotherapy; the anti-tumor capacities of CD8^+^ T cells derived from T_CM_ subsets remain still controversial and poorly understood [43, 44]. Future studies will be needed to determine the role(s) played by expansion kinetics of various T cell subsets with respect to long-term survival of patients undergoing CD19 CAR T cell therapy. In this study, we examined the expansion of circulating T_N_, T_CM_, T_EM_, and T_E_ subsets over time after CAR T cell infusion. Significantly lower percentages of circulating CD8^+^ T_N_ cells were detected in patients who ultimately relapsed after CD19 CAR T cell infusion when compared to those who experienced a long-term response. Equally important, T_CM_, T_EM_, and T_E_ subsets exhibited similar proliferation kinetics when comparing these two patient groups; these indicated that not all circulating T cell subsets were associated with disease relapse. Our findings suggest that CD8^+^ T_N_ cells may be the most effective subset to target when generating new adoptive immunotherapy strategies. The percentage of CD8^+^ T_N_ cells detected shortly after infusion may serve as a biomarker for long-term prognosis, as this is an independent factor associated with a shorter period of RFS. Interestingly, long-term responders persistently exhibited a more pronounced expansion of CD8^+^ T_N_ cells after infusion, although no significant differences with respect to the percentages of CD8^+^ T_N_ cells were detected at any other time points. A more definitive answer with respect to the role of CD8^+^ T_N_ cell percentages at other time points and their role in predicting a long-lasting response will likely require further research, especially as has become increasingly evident, that the endogenous immune system might have direct impact on the CAR T cells administered to patients [21, 45].

## Conclusions

The results presented in our study indicate that CD19 CAR T cells derived from hybridoma clone HI19α (HI19α-4-1BB-ζ CAR T) are highly effective against malignant cells in patients diagnosed with R/R B-ALL. Our results also suggest the percentage of circulating CD8^+^ T_N_ cells may be developed as a biomarker to predict the long-term prognosis of patients undergoing CAR T cell therapy.

## Supplementary information

**Additional file 1: Supplementary Table 1.** Antibodies used for flow cytometry staining of single cell suspensions. **Supplementary Table 2.** Computational alanine scanning on the complex of CD19 ECD and HI19α scFv. **Supplementary Table 3.** Computational alanine scanning on the complex of CD19 ECD and FMC63 scFv. **Supplementary Table 4.** Non-bond interaction between HI19α and hCD19 ECD. **Supplementary Table 5.** Non-bond interaction between FMC63 and hCD19 ECD. **Supplementary Table 6.** Univariate analysis for relapse-free survival. **Supplementary Table 7.** Multivariate analysis for relapse-free survival.

**Additional file 2: Supplementary Fig. 1.** Procedure of clinical trial and study flow of all participants. a Scheme for CAR T preparation and treatment. b The diagram shows all study participant’s course from the time of consent to treatment on study. **Supplementary Fig. 2.** Analysis for amplification of different T cell subpopulations after CD19 CAR T cell infusion. a–f, CD8^+^ T_CM_ cells (a), CD4^+^ T_CM_ cells (b), CD8^+^ T_EM_ cells (c), CD4^+^ T_EM_ cells (d), CD8^+^ T_E_ cells (e), CD4^+^ T_E_ cells (f) percentage in peripheral blood after CAR T cell infusion in patients with continuous CR or relapse from B-ALL. **Supplementary Fig. 3.** The expansion kinetics of Treg cells, NK-like T cells, and NK cells after CD19 CAR T cell infusion. a The correlation between CD19 CAR T cell expansion after infusion and the proliferation of Treg cells. b CD3^+^CD16^+^CD56^+^ NK-like T cells or CD3^-^CD16^+^CD56^+^ NK cells expansion in peripheral blood expansion after CAR T cell infusion. **Supplementary Fig. 4.** Analysis for amplification of CD19^+^ B cells according to relapse. a CD19^+^ B cells percentage in peripheral blood after CD19 CAR T cell infusion in patients with continuous CR. b CD19^+^ B cells percentage in peripheral blood after CD19 CAR T cell infusion in patients who relapsed from B-ALL.

## Data Availability

The datasets used during the current study are available from the corresponding author on reasonable request.

## Abbreviations

CAR T cell: Chimeric antigen receptor-modified T cell
scFv: Single-chain variable fragment
R/R B-ALL: Relapsed/refractory acute lymphoblastic leukemia
hCD19: Human CD19
ECD: Extracellular domain
Ph: Philadelphia chromosome
CNSL: Central nervous system leukemia
Kg: Kilogram
BM: Bone marrow
HSCT: Hematopoietic stem cell transplantation
Tregs: Regulatory T cells
T_N_: naïve T cells
T_CM_: Central memory T cells
T_EM_: Effector memory T cells
T_E_: Effector T cells
CR/CRi: Complete remission or complete remission with incomplete count recovery
MRD: Minimal residual disease
OS: Overall survival
RFS: Relapse-free survival
AE: Adverse event
CI: Confidence interval
NCCN: National Comprehensive Cancer Network
MSKCC: Memorial Sloan Kettering Cancer Center
NCI-CTCAE: National Cancer Institute Common Terminology Criteria for Adverse Events
CRS: Cytokine release syndrome
CRES: CAR T cell-related encephalopathy syndrome
