# Supplementary material for: Efficacy and safety of CD19 CAR T constructed with a new anti-CD19 chimeric antigen receptor in relapsed or refractory acute lymphoblastic leukemia
Source: J Hematol Oncol. 2020 Sep 7;13:122. doi: 10.1186/s13045-020-00953-8 (PMC7487702; doi:10.1186/s13045-020-00953-8)
Supplement: Supplementary file 1 — Additional file 1: Supplementary Table 1. Antibodies used for flow cytometry staining of single cell suspensions. Supplementary Table 2. Computational alanine scanning on the complex of CD19 ECD and HI19α scFv. Supplementary Table 3. Computational alanine scanning on the complex of CD19 ECD and FMC63 scFv. Supplementary Table 4. Non-bond interaction between HI19α and hCD19 ECD. Supplementary Table 5. Non-bond interaction between FMC63 and hCD19 ECD. Supplementary Table 6. Univariate analysis for relapse-free survival. Supplementary Table 7. Multivariate analysis for relapse-free survival. [file 13045_2020_953_MOESM1_ESM.pdf]

Supplementary Table 1. Antibodies used for flow cytometry staining of single cell suspensions

Antigen	Antibody clone	Supplier	Catalog number	Fluorophore
CD19	FMC63	Novus Biologicals	NBP2-52716	Unconjugated
CD19	HI19α	Biolegend	302254	PE
CD8	B9.11	Beckman Coulter	IM0451U	FITC
CD4	SK3	BD Biosciences	341654	PerCP/Cy5.5
CCR7	150503	BD Biosciences	560765	PE
CD3	UCHT1	BD Biosciences	558117	Pacific Blue
CD45RA	L48	BD Biosciences	337167	PE/Cy7
CD25	B1.49.9	Beckman Coulter	A07774	PE
CD127	A019D5	Biolegend	351316	APC
Anti-mouse IgG (H+L), F(ab')2 Fragment		Cell Signaling Technology	4410s	Alexa Fluor 647

Supplementary Table 2. Computational alanine scanning on the complex of CD19 ECD and HI19α scFv

Mutation	Mutation Energy	Effect of Mutation
C:LEU112>ALA	1.88	DESTABILIZING
C:GLY110>ALA	1.69	DESTABILIZING
C:PRO134>ALA	1.41	DESTABILIZING
C:GLU96>ALA	1.01	DESTABILIZING
C:GLN162>ALA	0.95	DESTABILIZING
C:LEU164>ALA	0.86	DESTABILIZING
C:GLY111>ALA	0.74	DESTABILIZING
C:PRO172>ALA	0.59	DESTABILIZING

Supplementary Table 3. Computational alanine scanning on the complex of CD19 ECD and FMC63 scFv

Mutation	Mutation Energy	Effect of Mutation
C:TRP177>ALA	3.29	DESTABILIZING
C:TRP61>ALA	2.37	DESTABILIZING
C:ARG56>ALA	2.09	DESTABILIZING
C:LEU58>ALA	1.63	DESTABILIZING
C:TRP91>ALA	1.39	DESTABILIZING
C:LEU164>ALA	1.32	DESTABILIZING
C:ASP218>ALA	1.23	DESTABILIZING
C:ARG230>ALA	1.15	DESTABILIZING
C:TRP220>ALA	0.94	DESTABILIZING

C:PHE63>ALA	0.84	DESTABILIZING
C:LEU168>ALA	0.84	DESTABILIZING
C:ASP158>ALA	0.8	DESTABILIZING
C:PRO183>ALA	0.71	DESTABILIZING
C:TRP261>ALA	0.67	DESTABILIZING
C:HIS54>ALA	0.65	DESTABILIZING
C:LEU160>ALA	0.52	DESTABILIZING
C:MET170>ALA	0.52	DESTABILIZING

Supplementary Table 4. Non-bond interaction between HI19α and hCD19 ECD

Name	Distance	Category	Types
C:LYS130:HZ1-H:ASP197:OD2	2.99968	Hydrogen Bond; Electrostatic	Salt Bridge;Attractive Charge
C:LYS117:NZ-H:ASP197:OD2	4.74657	Electrostatic	Attractive Charge
C:LYS130:NZ - H:ASP195:OD2	4.75474	Electrostatic	Attractive Charge
C:GLN162:HE21 - H:TYR60:O	2.87565	Hydrogen Bond	Conventional Hydrogen Bond
C:GLN162:HE22 - H:SER61:O	2.06008	Hydrogen Bond	Conventional Hydrogen Bond
C:THR169:HG1 - H:TYR60:OH	1.86865	Hydrogen Bond	Conventional Hydrogen Bond
H:TYR64:HH - C:SER163:O	2.42486	Hydrogen Bond	Conventional Hydrogen Bond
H:TYR64:HH - C:ASP167:O	2.32071	Hydrogen Bond	Conventional Hydrogen Bond
H:SER67:HG - C:PRO172:O	2.00899	Hydrogen Bond	Conventional Hydrogen Bond
H:SER78:HG - C:ASN161:O	2.76553	Hydrogen Bond	Conventional Hydrogen Bond
H:TYR172:HH - C:GLY97:O	2.46944	Hydrogen Bond	Conventional Hydrogen Bond
H:ARG238:HH21 - C:GLU96:OE1	2.56241	Hydrogen Bond	Conventional Hydrogen Bond
H:ARG238:HH22 - C:GLU96:OE1	2.46999	Hydrogen Bond	Conventional Hydrogen Bond
H:SER242:HG - C:VAL95:O	2.8557	Hydrogen Bond	Conventional Hydrogen Bond
H:SER242:HG - C:GLU96:O	3.02546	Hydrogen Bond	Conventional Hydrogen Bond
H:TYR248:HH - C:GLU96:O	2.22702	Hydrogen Bond	Conventional Hydrogen Bond
H:TYR251:HH - C:GLU96:OE2	2.18266	Hydrogen Bond	Conventional Hydrogen Bond
C:LYS130:HE2 - H:ASP195:OD2	2.74364	Hydrogen Bond	Carbon Hydrogen Bond
C:PRO134:HD1 - H:ILE241:O	3.0158	Hydrogen Bond	Carbon Hydrogen Bond
C:LEU164:HA - H:TYR64:OH	2.12798	Hydrogen Bond	Carbon Hydrogen Bond
C:MET170:HA - H:ARG65:O	2.29093	Hydrogen Bond	Carbon Hydrogen Bond
H:THR42:HB - C:ASN161:O	2.84799	Hydrogen Bond	Carbon Hydrogen Bond
H:SER67:HB2 - C:ALA171:O	2.02977	Hydrogen Bond	Carbon Hydrogen Bond
H:GLY194:HA2 - C:GLY129:O	3.08142	Hydrogen Bond	Carbon Hydrogen Bond
H:THR240:HB - C:GLU96:O	3.01836	Hydrogen Bond	Carbon Hydrogen Bond
H:SER242:HB2 - C:GLU96:O	1.96151	Hydrogen Bond	Carbon Hydrogen Bond
C:THR169:HN - H:TYR64	2.94094	Hydrogen Bond	Pi-Donor Hydrogen Bond
C:PRO134 - H:ILE241	4.84992	Hydrophobic	Alkyl
C:PRO134 - H:VAL244	4.05062	Hydrophobic	Alkyl
H:VAL244 - C:LEU101	5.48013	Hydrophobic	Alkyl
H:VAL244 - C:LEU116	3.88092	Hydrophobic	Alkyl

H:VAL245 - C:LEU112	5.05273	Hydrophobic	Alkyl
H:TYR64 - C:LEU168	5.37667	Hydrophobic	Pi-Alkyl
H:TYR192 - C:LYS130	5.49221	Hydrophobic	Pi-Alkyl

Supplementary Table 5. Non-bond interaction between FMC63 and hCD19 ECD

Name	Distance	Category	Types
F:ARG53:NE - C:ASP218:OD2	4.97651	Electrostatic	Attractive Charge
C:ARG56:HH11 - F:GLY247:O	2.06903	Hydrogen Bond	Conventional Hydrogen Bond
C:ARG56:HH12 - F:TYR246:O	2.05558	Hydrogen Bond	Conventional Hydrogen Bond
C:ARG56:HH12 - F:GLY248:O	2.07919	Hydrogen Bond	Conventional Hydrogen Bond
C:ARG56:HH22 - F:GLY248:O	2.54991	Hydrogen Bond	Conventional Hydrogen Bond
C:THR169:HN - F:SER198:O	2.17681	Hydrogen Bond	Conventional Hydrogen Bond
C:ARG230:HH12 - F:LEU54:O	2.05587	Hydrogen Bond	Conventional Hydrogen Bond
F:TYR32:HH - C:PRO57:O	1.97098	Hydrogen Bond	Conventional Hydrogen Bond
F:TYR204:HH - C:SER159:O	2.09663	Hydrogen Bond	Conventional Hydrogen Bond
F:SER210:HG - C:ASP158:OD1	2.59296	Hydrogen Bond	Conventional Hydrogen Bond
F:THR213:HG1 - C:ASP158:O	1.99273	Hydrogen Bond	Conventional Hydrogen Bond
C:HIS54:HD2 - F:GLY247:O	1.96267	Hydrogen Bond	Carbon Hydrogen Bond
C:ARG56:HD2 - F:GLY247:O	3.00059	Hydrogen Bond	Carbon Hydrogen Bond
C:LEU164:HA - F:GLU199:O	2.0872	Hydrogen Bond	Carbon Hydrogen Bond
C:LEU168:HA - F:SER198:O	1.98845	Hydrogen Bond	Carbon Hydrogen Bond
F:SER198:HA - C:THR169:O	2.15073	Hydrogen Bond	Carbon Hydrogen Bond
F:THR201:HA - C:LEU164:O	2.24241	Hydrogen Bond	Carbon Hydrogen Bond
F:SER210:HA - C:ASP158:OD1	2.14936	Hydrogen Bond	Carbon Hydrogen Bond
F:THR213:HA - C:ASP158:O	2.71305	Hydrogen Bond	Carbon Hydrogen Bond
F:ILE215:HA - C:ASN161:OD1	2.47184	Hydrogen Bond	Carbon Hydrogen Bond
C:ARG56:NE - F:TYR251	4.30386	Electrostatic	Pi-Cation
C:ARG56:HH21 - F:TYR251	2.16168	Hydrogen Bond	Pi-Donor Hydrogen Bond
C:TRP177 - F:TRP195	5.42153	Hydrophobic	Pi-Pi Stacked
C:TRP177 - F:TRP195	4.34988	Hydrophobic	Pi-Pi Stacked
C:TRP177 - F:TRP195	4.01551	Hydrophobic	Pi-Pi Stacked
F:TRP195 - C:TRP177	4.5345	Hydrophobic	Pi-Pi Stacked
C:PRO183 - F:LEU96	4.28572	Hydrophobic	Alkyl
C:PRO184 - F:LEU96	4.52884	Hydrophobic	Alkyl
F:TYR32 - C:PRO57	4.90288	Hydrophobic	Pi-Alkyl
F:HIS50 - C:PRO57	5.19082	Hydrophobic	Pi-Alkyl
F:TYR203 - C:VAL182	5.28128	Hydrophobic	Pi-Alkyl
F:TYR203 - C:PRO183	4.73926	Hydrophobic	Pi-Alkyl

Supplementary Table 6. Univariate analysis for relapse-free survival

	B	SE	Wald	HR	*P*	95% CI
$CD8^+$ T_N (Q2-4 /Q1) *	-1.940	0.781	6.169	0.144	0.013	0.031-0.664

$CD4^+$ T_N (Q2-4 /Q1) *	-0.892	0.676	1.743	0.410	0.187	0.109-1.541
Age (≥18/<18 years old)	1.301	0.684	3.623	3.674	0.057	0.962-14.03
High-risk gene mutation or deletion *(yes/no)	0.141	0.628	0.050	1.151	0.823	0.336-3.938
Glucocorticoid therapy (yes/no)	-0.496	0.694	0.510	0.609	0.475	0.156-2.375
Transplantation (yes/no)	-1.919	0.688	7.789	0.147	0.005	0.038-0.565

T_N, naïve T cells; Q2-4 and Q1, $CD8^+$ T_N cells percentage in 20 min after CD19 CAR T infusion were ranked and divided the into four quartiles, with the lowest $CD8^+$ T_N cells frequency in quartile one (Q1) and the greatest in quartile four (Q4); High-risk gene mutation or deletion include T315I mutation, TP53 mutation, IKZF1 deletion

Supplementary Table 7. Multivariate analysis for relapse-free survival

	**B**	**SE**	**Wald**	**HR**	***P***	**95% CI**
$CD8^+$ T_N (Q2-4 /Q1) *	-2.279	0.903	6.373	0.102	0.012	0.017-0.601
Transplantation (yes/no)	-1.213	0.928	1.710	0.297	0.191	0.048-1.832
Age (≥18/<18 years old)	0.814	0.876	0.864	2.257	0.353	0.406-12.56

T_N, naïve T cells; Q2-4 and Q1, $CD8^+$ T_N cells percentage in 20 min after CD19 CAR T infusion were ranked and divided the into four quartiles, with the lowest $CD8^+$ T_N cells frequency in quartile one (Q1) and the greatest in quartile four (Q4)
