# Supplementary material for: Efficacy and safety of CD19 CAR T constructed with a new anti-CD19 chimeric antigen receptor in relapsed or refractory acute lymphoblastic leukemia
Source: J Hematol Oncol. 2020 Sep 7;13:122. doi: 10.1186/s13045-020-00953-8 (PMC7487702; doi:10.1186/s13045-020-00953-8)
Supplement: Supplementary file 2 — Additional file 2: Supplementary Fig. 1. Procedure of clinical trial and study flow of all participants. a Scheme for CAR T preparation and treatment. b The diagram shows all study participant’s course from the time of consent to treatment on study. Supplementary Fig. 2. Analysis for amplification of different T cell subpopulations after CD19 CAR T cell infusion. a–f, CD8+ TCM cells (a), CD4+ TCM cells (b), CD8+ TEM cells (c), CD4+ TEM cells (d), CD8+ TE cells (e), CD4+ TE cells (f) percentage in peripheral blood after CAR T cell infusion in patients with continuous CR or relapse from B-ALL. Supplementary Fig. 3. The expansion kinetics of Treg cells, NK-like T cells, and NK cells after CD19 CAR T cell infusion. a The correlation between CD19 CAR T cell expansion after infusion and the proliferation of Treg cells. b CD3+CD16+CD56+ NK-like T cells or CD3-CD16+CD56+ NK cells expansion in peripheral blood expansion after CAR T cell infusion. Supplementary Fig. 4. Analysis for amplification of CD19+ B cells according to relapse. a CD19+ B cells percentage in peripheral blood after CD19 CAR T cell infusion in patients with continuous CR. b CD19+ B cells percentage in peripheral blood after CD19 CAR T cell infusion in patients who relapsed from B-ALL. [file 13045_2020_953_MOESM2_ESM.pdf]

Fig. S1

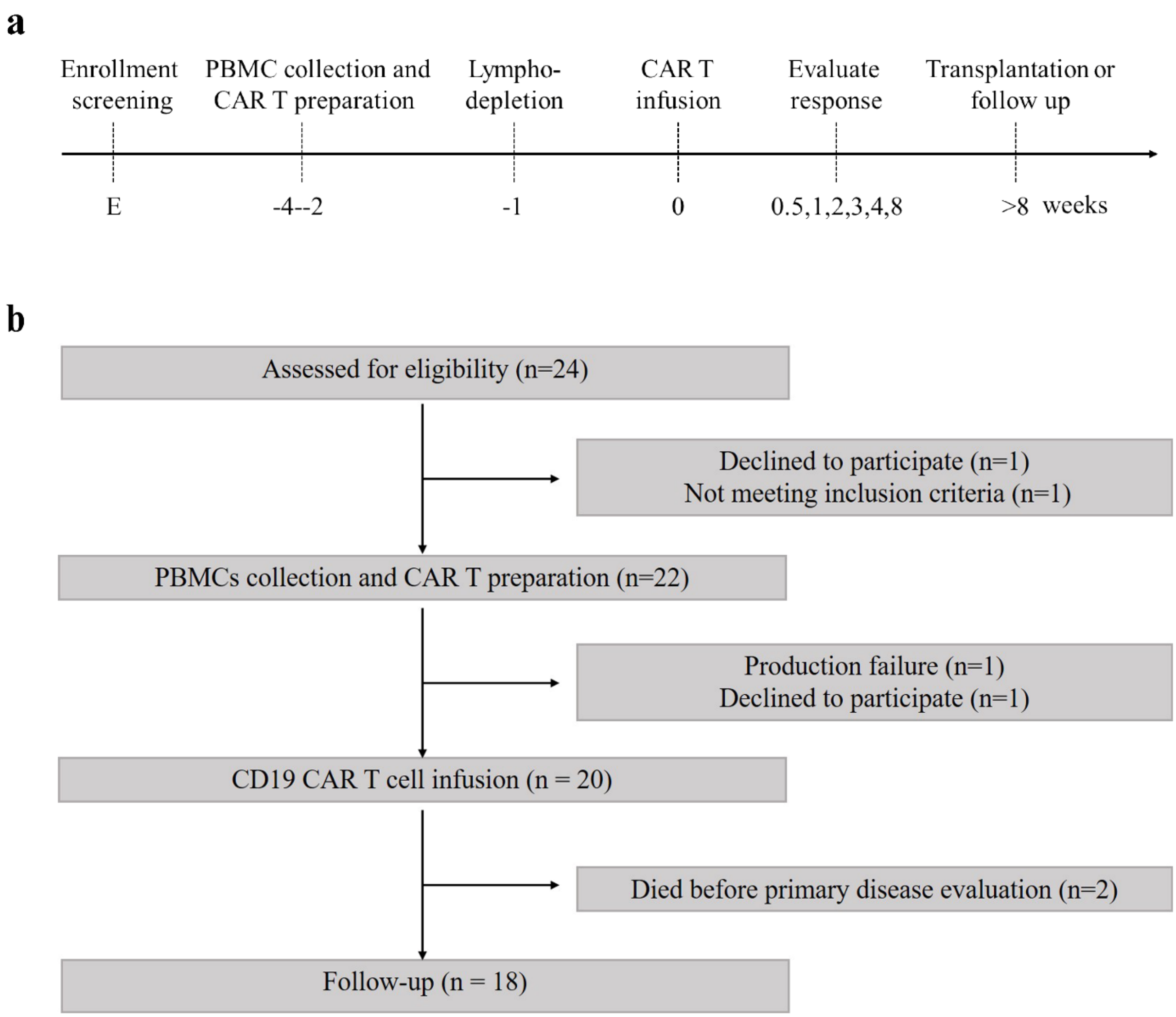

Supplementary Fig. 1 Procedure of clinical trial and study flow of all participants

Supplementary Fig. 1 legends a. Scheme for CAR T preparation and treatment. b. The diagram shows all study participant’s course from the time of consent to treatment on study.

Fig. S2

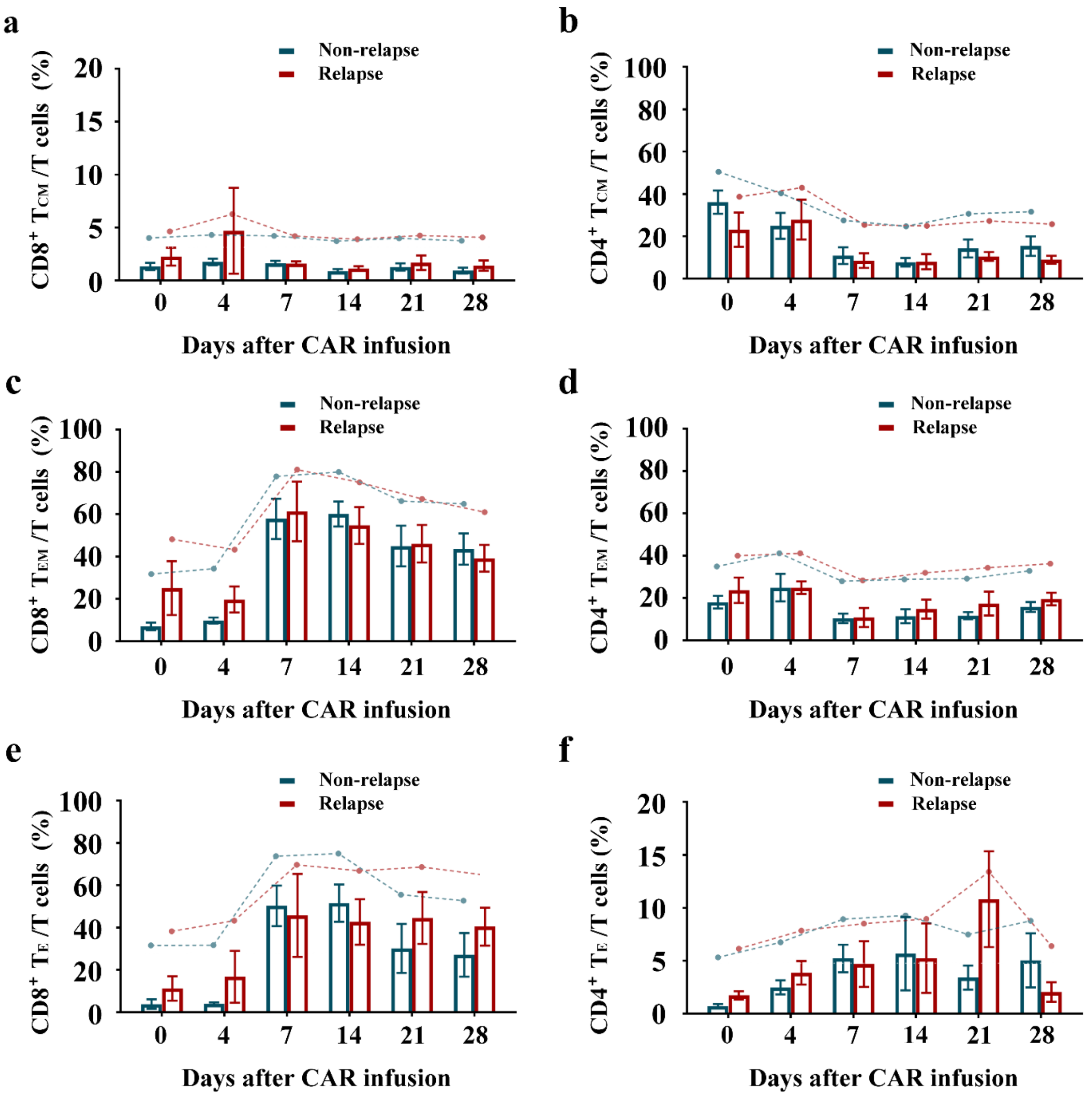

Supplementary Fig. 2 Analysis for amplification of different T cell subpopulations after CD19 CAR T cell infusion

Supplementary Fig. 2 legends. a-f, $CD8^+$ T_{CM} cells (a), $CD4^+$ T_{CM} cells (b), $CD8^+$ T_{EM} cells (c), $CD4^+$ T_{EM} cells (d), $CD8^+$ T_E cells (e), $CD4^+$ T_E cells (f) percentage in peripheral blood after CAR T cell infusion in patients with continuous CR or relapse from B-ALL.

Fig. S3

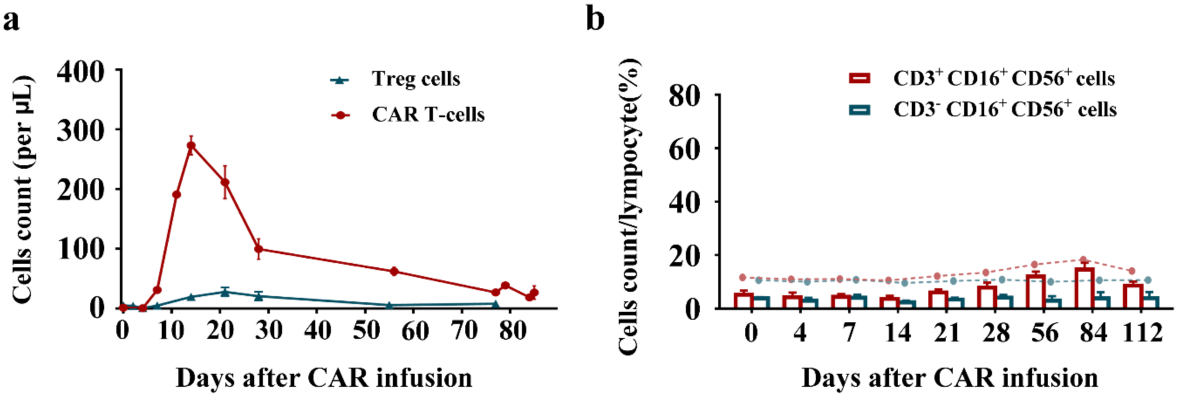

Supplementary Fig. 3 The expansion kinetics of Treg cells, NK-like T cells and NK cells after CD19 CAR T cell infusion

Supplementary Fig. 3 legends. a. The correlation between CD19 CAR T cell expansion after infusion and the proliferation of Treg cells. b. $CD3^{+}CD16^{+}CD56^{+}$ NK-like T cells or $CD3^{-}CD16^{+}CD56^{+}$ NK cells expansion in peripheral blood expansion after CAR T cell infusion.

Fig. S4

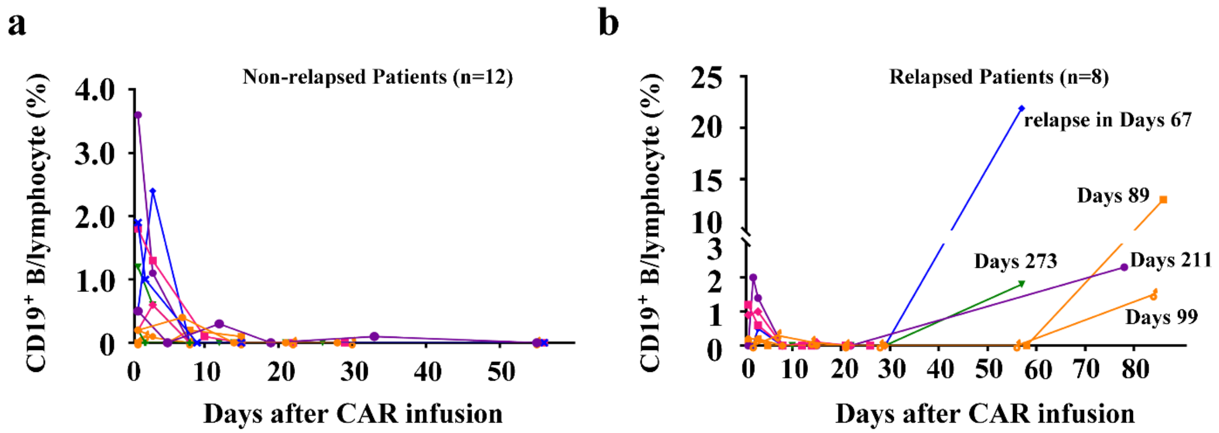

Supplementary Fig. 4 Analysis for amplification of $CD19^{+}$ B cells according to relapse

Supplementary Fig. 4 legends. a. $CD19^{+}$ B cells percentage in peripheral blood after CD19 CAR T cell infusion in patients with continuous CR. b. $CD19^{+}$ B cells percentage in peripheral blood after CD19 CAR T cell infusion in patients who relapsed from B-ALL.
